# Cystic Fibrosis: Recent Insights into Inhaled Antibiotic Treatment and Future Perspectives

**DOI:** 10.3390/antibiotics10030338

**Published:** 2021-03-22

**Authors:** Giovanni Taccetti, Michela Francalanci, Giovanna Pizzamiglio, Barbara Messore, Vincenzo Carnovale, Giuseppe Cimino, Marco Cipolli

**Affiliations:** 1 Cystic Fibrosis Center, Anna Meyer Children’s University Hospital, Viale Pieraccini 24, 50139 Firenze, Italy; michela.francalanci@meyer.it; 2 Department Pathophysiology and Transplantation, University of Milan, IRCCS Fondazione Ca’ Granda Ospedale Maggiore Policlinico, Via Francesco Sforza 35, 20122 Milan, Italy; giovanna.pizzamiglio@policlinico.mi.it; 3 Adult Cystic Fibrosis Center, Azienda Ospedaliera Universitaria San Luigi Gonzaga, 10043 Orbassano, Italy; b.messore@sanluigi.piemonte.it; 4 Adult Cystic Fibrosis Center, Department of Translational Medical Sciences, Federico II University of Naples, Via Sergio Pansini 5, 80131 Naples, Italy; vincenzo.carnovale@unina.it; 5 Cystic Fibrosis Center, Policlinico Umberto I Hospital, Viale Regina Elena 324, 00161 Rome, Italy; gi.cimino@policlinicoumberto1.it; 6 Cystic Fibrosis Center, Azienda Ospedaliero Universitaria Integrata di Verona, Pl. Aristide Stefani 1, 37126 Verona, Italy; marco.cipolli@aovr.veneto.it

**Keywords:** cystic fibrosis, *P. aeruginosa*, inhaled antibiotics, pulmonary exacerbations

## Abstract

Although new inhaled antibiotics have profoundly improved respiratory diseases in cystic fibrosis (CF) patients, lung infections are still the leading cause of death. Inhaled antibiotics, i.e., colistin, tobramycin, aztreonam lysine and levofloxacin, are used as maintenance treatment for CF patients after the development of chronic *Pseudomonas aeruginosa* (*P. aeruginosa*) infection. Their use offers advantages over systemic therapy since a relatively high concentration of the drug is delivered directly to the lung, thus, enhancing the pharmacokinetic/pharmacodynamic parameters and decreasing toxicity. Notably, alternating treatment with inhaled antibiotics represents an important strategy for improving patient outcomes. The prevalence of CF patients receiving continuous inhaled antibiotic regimens with different combinations of the anti-*P. aeruginosa* antibiotic class has been increasing over time. Moreover, these antimicrobial agents are also used for preventing acute pulmonary exacerbations in CF. In this review, the efficacy and safety of the currently available inhaled antibiotics for lung infection treatment in CF patients are discussed, with a particular focus on strategies for eradicating *P. aeruginosa* and other pathogens. Moreover, the effects of long-term inhaled antibiotic therapy for chronic *P. aeruginosa* infection and for the prevention of pulmonary exacerbations is reviewed. Finally, how the mucus environment and microbial community richness can influence the efficacy of aerosolized antimicrobial agents is discussed.

## 1. Introduction

Cystic fibrosis (CF) is characterized by chronic infection, inflammation of the airways and a progressive decline in lung function [1]. Although patients with CF experience multiple bacterial infections throughout their lives, the most common pathogens responsible for respiratory tract infections are *Staphylococcus aureus* and *P.aeruginosa* [2]. Respiratory syncytial virus (RSV) infection has been shown to promote *P. aeruginosa* colonization [3,4]. Moreover, methicillin-resistant *Staphylococcus aureus* (MRSA) infection has emerged as a potentially harmful pathogen in CF since persistent infection due to this bacterium is associated with reduced lung function and higher mortality [5,6,7,8,9].

The lifespan of CF patients has risen markedly over the past four decades [10]. Although improvements in survival have been achieved by adopting a multidisciplinary team approach in CF care centers [11], the vast majority of patients still die from respiratory failure [12,13]. Since lung disease is the major source of CF-related complications, the primary target of antibiotic therapy is to preserve or improve, whenever possible, lung function by means of meticulous daily management of the pulmonary disease, together with the prompt and intensive treatment of pulmonary exacerbations [14]. Changes in antimicrobial treatment or in continuous antibiotic regimens are also made to prevent and reduce deterioration in lung function before the insurgence of pulmonary exacerbations [8,15,16]. Although the recent introduction of novel therapies targeting the defective CF transmembrane conductance regulator (CFTR) protein has transformed the landscape of CF [17,18], the antibiotic treatment of respiratory infections will remain the mainstay of CF therapy for the foreseeable future [19,20]. Accordingly, new treatment strategies for better management of acute and chronic episodes have been at the forefront of efforts to improve outcomes in CF. The entry of specific formulated preparations for aerosol administration onto the market, accompanied by the improved performance of inhalational drug delivery devices and the anti-inflammatory effects of azithromycin, has profoundly changed the concept of chronic suppressive therapy in CF. Forced expiratory volume in one second (FEV_1_) is an established marker of CF disease progression, which is used to capture clinical course and evaluate the therapeutic efficacy of antimicrobial treatments [21].

The formation of a biofilm supports the persistence of *P. aeruginosa* and can also contribute to the pathogenesis of pulmonary exacerbations [22]. In this regard, the impaired penetration of antimicrobials through the bacterial biofilm is considered to be one of the most important reasons for the failure of anti-pseudomonal therapy [23,24]. However, recent studies have provided evidence for a highly complex microbiome in the CF airway which includes not only the “traditional” CF pulmonary pathogens, but also other microorganisms [25,26].

A better understanding of the airway microbiota and host–pathogen interactions in CF disease is believed to be decisive in improving the management of CF lung infections and in tailoring appropriate antibiotic treatments. 

In this review, the currently available inhaled antibiotics for the management of lung infections in CF patients are discussed, with a particular focus on the eradication of *P. aeruginosa* infection, and the effects of inhaled antibiotic therapy for chronic *P. aeruginosa* infection as well as for preventing pulmonary exacerbations. 

The review provides a forward-looking perspective in regard to the impact of the mucus environment on *P. aeruginosa* biofilm formation and the airway microbial community composition in terms of the efficacy of aerosolized antimicrobial agents in CF.

## 2. Rationale for Inhaled Antibiotics in CF

**Figure 1:**
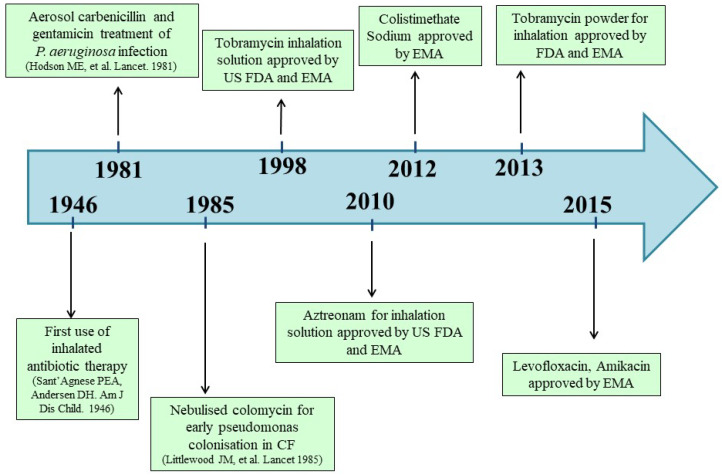
Timetable of the most relevant milestones in the evolution of inhaled antibiotic therapy in cystic fibrosis.

The rationale for inhaled antibiotics as a means of administering a targeted treatment to the lower respiratory tract was first introduced in 1946; however, it has only been consistently implemented over the past three decades [14,27] (Figure 1).

Currently, chronic inhaled antibiotic administration is the standard of care for the management of chronic *P. aeruginosa* infection in CF patients, based on significant evidence showing a decrease in the *P. aeruginosa* density in the sputum as well as an improvement in respiratory symptoms, quality of life and lung function [14,16].

Aerosolized antibiotics offer advantages over systemic therapy (oral or intravenous), since relatively high levels of the drug can be delivered directly to the airway, thus, improving the pharmacokinetic/pharmacodynamic indices and reducing systemic toxicity [14,28,29]. Daily inhaled antibiotics are used to reduce the risk of pulmonary exacerbations in CF adults with chronic *P. aeruginosa* infection [30]. Nonetheless, there are limited options of antibiotics that have been formulated and approved for inhaled use; since the effectiveness of antibiotics decreases over time, the development of alternative inhaled antimicrobial agents is desirable. At present, colistin, tobramycin, aztreonam lysine and, more recently, levofloxacin represent the most commonly used inhaled antibiotics for the management of CF infections (Figure 1, Table 1) [29,31,32,33].

In particular, inhaled colistin has been used extensively in Europe, while inhaled tobramycin is recommended in the USA to improve lung function and health-related quality of life, while decreasing the risk of exacerbation in CF patients chronically infected with *P. aeruginosa* [15]. Tobramycin also exhibits superior activity against *P. aeruginosa* as compared to other aminoglycosides. Nebulized aztreonam lysine was approved by the US Food and Drug Administration in 2010; its clinical efficacy and tolerability in patients with chronic *P. aeruginosa* infection have been proven in several clinical trials [34,35,36]. The clinical efficacy and tolerability of nebulized levofloxacin, a broad-spectrum, third-generation fluoroquinolone antibiotic, have been reported in a phase III clinical trial which included CF patients chronically infected with *P. aeruginosa* [33,37]. In particular, the CF patients who received inhaled levofloxacin showed a decreased sputum density of *P. aeruginosa*, an improvement in lung function parameters, and a reduction in exacerbation and hospitalization rates [33,38,39]. Other inhaled antibiotics, such as the fosfomycin/tobramycin inhalation solution and amikacin liposome inhalation suspension, are currently being introduced into CF treatment regimens [40,41]. Furthermore, there is significant interest in developing novel inhaled antibiotic treatments for CF-related pathogens other than *P. aeruginosa*. For example, the ongoing clinical trial NCT 01315236 is specifically designed to evaluate the effectiveness of inhaled vancomycin for the treatment of MRSA (AeroVanc; Savara Pharmaceuticals, Austin, TX, USA).

Despite the availability of several nebulized antimicrobials and treatment regimens for CF, there is no clear recommendation on how to select the most appropriate inhaled antibiotic based on different patient characteristics. As mentioned before, it has been proposed that continuous alternating treatment with inhaled antibiotics might be an important strategy to improve the clinical outcome of CF disease [16]. Regrettably, long-term efficacy data are unavailable, and the maximum length of the studies varies from 6 to 24 months [42]. Moreover, the long-term use of inhaled antibiotics, particularly of formulations of dry powder, can cause local side effects for some CF patients [28,43].

The clinical relevance of resistance to antibiotics administered via inhalation is still not completely understood. Antimicrobial susceptibility test results did not appear to influence antibiotic selection; rather, it was the clinical response which had the strongest influence on the decision to change antibiotic treatment [44]. The majority of studies regarding long-term inhaled antibiotics have failed to prove the occurrence of newly acquired resistance in people with CF [45,46]. This could be because the concentrations of antimicrobial agents delivered via inhalation to the lungs are above the conventional minimum inhibitory concentrations (MICs) used for systemic administration [47].

## 3. Current Status of Inhaled Antibiotics in Cystic Fibrosis

The clinical efficacy of the available inhaled antibiotics licensed in Europe (colistimethate sodium, tobramycin, aztreonam lysine and levofloxacin) and in the USA (tobramycin and aztreonam lysine) for the treatment of pulmonary infection caused by *P. aeruginosa* in CF patients are discussed below (Table 1).

### 3.1. Colistimethate Sodium

Colistimethate sodium (colistin), a cationic polypeptide, acts by disrupting the integrity of the bacterial cell membrane. Inhaled colistin has been used in *P. aeruginosa*-related CF therapy in Europe for decades, although no randomized, placebo-controlled trials have been conducted which favor its use [48]. In a perspective, double-blind, placebo-controlled study, colistin inhalation therapy was superior to placebo control in terms of a better clinical symptom score, and the preservation of lung function and inflammatory markers in CF patients [49]. Side effects related to airway reactivity are a frequent problem in aerosol therapy and have been reported following the use of inhaled colistin. The incidence of adverse events was comparable in CF patients who received a 4-week, twice-daily aerosol administration of either tobramycin nebulizer solution (TNS) or nebulized colistin [50]. Colistimethate therapy was also associated with the development of a fatal case of acute respiratory distress syndrome in a 29-year-old woman with CF; however, the antibiotic formulation was administered by aerosol after a prolonged reconstitution time [51]. More recently, colistimethate sodium has been produced as a dry powder formulation to be administered via a hand-held inhaler. Moreover, colistimethate sodium, delivered as a dry powder for inhalation, was not inferior to the tobramycin inhalation solution (TIS) in the treatment of CF patients 6 years of age and older with chronic *P. aeruginosa* infection, as evaluated by the change in percentage predicted FEV_1_ after 24 weeks [52]. The use of colistin-based dry powder in the treatment of CF patients is commonly associated with side effects, including a cough, oropharyngeal pain and abnormal taste; however, these side effects have been reduced by improving inhalation techniques [53]. Of note, the results obtained from a recent observational comparative cohort study using data from the UK Cystic Fibrosis Registry (UKCFR) from 1 January 2014 to 31 December 2018, in a real-world setting of CF patients aged 6 years or older treated with colistimethate sodium dry powder, showed that the safety profile of inhaled colistin was similar to that of other inhaled anti-pseudomonal antibiotics [54].

### 3.2. Tobramycin

Tobramycin is an aminoglycoside antibiotic which inhibits the initiation of protein synthesis by interfering with the formation of the initiation complex between mRNA and the bacterial small 30S ribosomal subunit. The intermittent administration of TIS improves lung function and decreases the density of *P. aeruginosa* in sputum specimens from CF patients along with the rates of hospitalization [55,56]. TIS therapy improved lung function and weight gain in CF patients (13–17 years of age) over a 2-year period of intermittent treatment [57]. Moreover, TIS was associated with tinnitus, alteration of the voice and transient increases in creatinine; however, long-term tobramycin aerosol therapy has been proven to be well tolerated with no unexpected adverse events in patients with CF [55]. In order to decrease the treatment burden of CF patients, an inhalation powder formulation of tobramycin (TIP), delivered via the T-326 Inhaler, has been used as an alternative for treating CF patients with *P. aeruginosa* infection [58]. Controlled clinical and real-world studies have shown that TIP, delivered via the T-326 Inhaler, had a safety and efficacy profile comparable to TIS, with greater treatment satisfaction and adherence rates [58]. Using azithromycin in combination with inhaled tobramycin was common in USA CF patients with chronic *P. aeruginosa* infection. However, recent data have indicated that azithromycin reduced the antibacterial activity of tobramycin by promoting adaptive bacterial stress responses in *P. aeruginosa* infected individuals [59].

### 3.3. Aztreonam Lysine

Aztreonam lysine, known as AZLI, is an aerosolized formulation of the monobactam antibiotic aztreonam and lysine. Aztreonam lysine inhibits bacterial cell wall synthesis by interacting with penicillin-binding protein 3; it shows activity against a number of Gram-negative bacteria, including *P. aeruginosa* [60]. The efficacy and safety of inhaled aztreonam lysine on maintenance treatment for *P. aeruginosa* infection has been demonstrated in a randomized, double-blind, placebo-controlled study [35]. In particular, AZLI treatment (75 mg three times daily for 28 days) improved mean respiratory function scores and reduced sputum *P. aeruginosa* density in CF patients after pretreatment with TIS [35]. Moreover, AZLI treatment enhanced the median time needed for additional antibacterial agents to treat respiratory symptoms of pulmonary exacerbations [35]. Similarly, a 28-day treatment regimen with AZLI was found to improve respiratory symptoms and lung function in CF patients with clinically moderate-to-severe lung disease [36]. In an open-label, parallel-group international trial, three cycles of inhaled AZLI (28 days on–28 days off) demonstrated higher clinical efficacy as compared to TIS by improving lung function and reducing the number of exacerbations [61]. On the other hand, no considerable improvement in respiratory symptoms was observed in CF patients over 6 years of age with mild lung disease receiving aztreonam for inhalation solution three times daily for 28 days with a 14-day follow-up [62]. Moreover, 24 weeks of continuous AZLI administration was shown to not ameliorate lung function in CF patients with chronic *Burkholderia cepacia* complex (Bcc) infection or other emerging pathogens [63]. The adverse events which are usually reported with AZLI therapy have been classified as mild or moderate. A cough is the most common side effect associated with this inhaled antibiotic agent. Adverse reactions in pediatric patients with CF were lower than those observed in adults [36,62].

### 3.4. Levofloxacin

Levofloxacin (LIS) is a broad-spectrum, third-generation fluoroquinolone antibiotic and optically active L-isomer of ofloxacin with antibacterial activity. It inhibits bacterial DNA gyrase and topoisomerase IV, thus, blocking bacterial cell growth. In several phase I studies, it has been observed that nebulized levofloxacin generates high sputum and low serum drug concentrations in stable CF patients, minimizing the risk of systemic toxicity [32]. Moreover, its administration in an aerosol formulation was well tolerated and resulted in a reduction in the *P. aeruginosa* load in the sputum at day 28, in an improvement in pulmonary function (such as a change in percentage predicted FEV_1_) and in a decrease in the use of other antipseudomonal agents [33]. Levofloxacin was superior to TIS in terms of mean FEV_1_ change from baseline to day 28 of the second and third treatment cycles (28 day on/off) in CF patients (≥18 years old) [64]. In addition, LIS has been shown to reduce the risk of pulmonary exacerbation over a 24-week study period in CF adults as compared to those treated with TIS [64]. This inhaled antibiotic, rather than trimethoprim–sulfamethoxazole (TMP/SMX) [65], might also be effective in treating the coinfection of *Stenotrophomonas maltophilia* and *P. aeruginosa*. A systematic literature review and in-depth Bayesian network meta-analysis was carried out on CF patients to achieve the following: (1) collect clinical evidence regarding the use of inhaled antibiotics for the management of chronic *P. aeruginosa* infection and (2) compare the short-term (4 weeks) and long-term (24 weeks) efficacy of levofloxacin as compared to other inhaled antibiotics. These analyses did not produce strong enough evidence to show that the other currently available inhaled antibiotics were more effective than LIS for the treatment of chronic CF *P. aeruginosa* lung infections [66]; LIS was safe and generally well-tolerated in CF patients. In a randomized trial, the safety profile of LIS was similar to that of TIS [37]. The most notable difference in the safety profiles was the higher incidence of dysgeusia in patients receiving LIS [37].

## 4. Antibiotic Strategies for Eradicating *P. aeruginosa* and Other Pathogenic Organisms

Upon initial *P. aeruginosa* isolation from the CF airway, patients are intensively treated (in the early stages) with antibiotics to eradicate the pathogen, prevent chronic colonization and its associated long-term adverse outcomes, such as lung function decline and earlier mortality [67]. Early eradication antibiotic therapy should be initiated promptly, possibly within 4 weeks from the first positive culture result for *P. aeruginosa* [13,14]. Several clinical trials have demonstrated the efficacy of rigorous antibiotic treatment in eradicating *P. aeruginosa* from airway secretions [68,69]. Hence, antibiotic eradication therapy for early *P. aeruginosa* infection is now considered the standard of care in patients with CF, although more evidence is needed to identify the most efficacious regime of antibiotic eradication therapy [68,69,70]. Despite the high intrapulmonary concentrations achievable with inhaled antibiotics (e.g., tobramycin), eradication fails in approximately 10–40% of CF patients colonized by *P. aeruginosa* [68,69,70,71,72,73,74,75,76]. Currently, it still remains unclear which inhaled antibiotic treatment option should be labelled as the “gold standard” for the eradication of *P. aeruginosa*. The results of a large, multicentric, randomized, placebo-controlled clinical study (OPTIMIZE (Optimizing Treatment for Early *Pseudomonas aeruginosa* Infection in Cystic Fibrosis)) carried out on CF children with an initial colonization of *P. aeruginosa* infection have shown that the addition of oral azithromycin to TIS decreased the risk of exacerbation and was associated with an improvement in weight [77]. However, there were no significant differences in microbiological outcomes [77]. Nebulized gentamicin solution combined with systemic antibiotics has been demonstrated to be safe and to have similar efficacy to other eradicating therapies for new *P. aeruginosa* infection in young CF patients [78]. More recently, a multicentric, parallel group, randomized controlled trial was conducted to compare the efficacy of intravenous treatment with ceftazidime and tobramycin versus oral ciprofloxacin in the eradication of early *P. aeruginosa* infection. In both regimens, inhaled colistimethate sodium was given concurrently for three months. Parenteral treatment was not more effective than oral ciprofloxacin therapy for the eradication of *P. aeruginosa* [79]. 

Along with inhaled antibiotic strategies for eradicating *P. aeruginosa* in CF patients, novel therapeutic protocols have been proposed for the eradication of MRSA or emerging Bcc pathogens. Numerous protocols for the early eradication of MRSA have been reported to prevent the negative clinical implications of MRSA colonization [80]. A recent systemic review published by Lo et al. examined two trials with MRSA-infected patients; in both, the active treatment was oral trimethoprim and sulfamethoxazole associated with rifampicin, while the control arm was observational only [81]. From this study, it emerged that it was possible to eradicate MRSA from the airways of CF patients, with one trial showing a higher efficacy for the active MRSA treatment as compared with the observation-only control arm in terms of the percentage of patients with MRSA negative cultures at day 28. However, after six months, the number of patients with CF who remained MRSA-free was similar between the treatment arms in both trials [81]. Despite this evidence, some new MRSA eradication protocols have recently been proposed. In this scenario, successful MRSA eradication was achieved in 86% of the CF patients treated with oral rifampicin and fusidic acid, inhaled vancomycin, intranasal mupirocin, and following hygienic directives over a 7-day period [82]. An additional study was carried out to examine the efficacy of an eradication treatment protocol against new-onset MRSA infection with oral rifampicin and trimethoprim/sulfamethoxazole. This lasted 21 days and involved a 2% nasal mupirocin—each nostril three times daily for 5 days (active arm)—in CF patients enrolled in different clinical centers in Italy [83]. This study reported 24.7% higher clearance of MRSA for CF patients in the active arm as compared to those in the observational arm at 6 months, confirming previous data [81,84,85,86,87,88]. In another study, it was found that the use of a single course of inhaled vancomycin was not associated with increased rates of MRSA eradication in CF patients and may have led to bronchospasms [89]. Moreover, longitudinal studies have reported different microbiological follow-up durations, highlighting the difficulty of drawing any definitive conclusions regarding MRSA eradication therapies [81]. 

Other respiratory bacteria, such as species of the *Burkholderia cepacia* complex, can adapt to the lung environment of CF patients, although with a lower prevalence than *P. aeruginosa*. Infection with Bcc results in a heterogeneous clinical outcome, ranging from the asymptomatic colonization of the respiratory tract to chronic infections associated with decreased lung function and enhanced mortality in CF patients. Thus, it is essential to determine whether an inhaled antibiotic treatment protocol in eradicating Bcc infection can be developed; however, to date, there is still insufficient evidence for guiding treatment protocols. Regarding this issue, the clearance of the Bcc from sputum cultures, using an aggressive combination of intravenous, inhaled and oral antibiotic regimens, including an induction (tobramycin, ceftazidime, trimethoprim/sulfamethoxazole, inhaled tobramycin, azithromycin) and a consolidation (trimethoprim/sulfamethoxazole, inhaled tobramycin, azithromycin) period was effective after one year of treatment and was associated with clinically stable CF lung disease [90]. In fact, a recent systemic review pointed out that there is a lack of evidence regarding a safe and effective therapeutic strategy for Bcc eradication in people with CF, suggesting an urgent need to carry out well-designed multicentric randomized controlled trials on a variety of novel antibiotics [91].

In addition to traditional pathogens, other bacteria such as the *Achromobacter* species, are increasingly being detected in respiratory samples from CF patients [92]. Notably, it has been shown that early treatment with inhaled antibiotics consisting of inhaled ceftazidime, colistin or tobramycin may eradicate infection with *Achromobacter* in CF patients [93].

## 5. Inhaled Antibiotic Therapy for *P. aeruginosa* Chronic Pulmonary Infection

It has been well established that many patients with CF are chronically infected with *P. aeruginosa*, which is considered to be one of the major drivers of lung disease. Suppressive therapy with inhaled antibiotics improves lung function and reduces the rate of pulmonary exacerbation; however, more evidence is needed from trials to reach definitive conclusions [8]. According to a systemic review on inhaled anti-pseudomonal antibiotics for long-term therapy in CF, there are limited data available for carrying out meta-analyses due to the variation across studies or heterogeneity in the study results [8]. There are also scarce data regarding the effects of inhaled antimicrobial agents on nutritional outcomes or survival, as well as on quality of life [8].

Approved inhaled antibiotics (such as aztreonam and tobramycin) have traditionally been used in a 28-day intermittent treatment strategy (also known as the “on/off” regimen), which was based upon the observation that there was a slight added benefit to pulmonary function following 4 weeks of antimicrobial treatment, and the statement that intermittent antibiotic use would reduce the bacterial density without selecting resistant bacteria [55,94]. It now appears that a clinical response occurs even when antibiotic resistant bacteria are detected in the sputum cultures and that the loss of the lung function benefit due to antibiotic treatment occurs during the “off” periods [41,55,61,95,96].

There is a growing trend towards the use of continuous inhaled antibiotic therapy in CF, either as monotherapy with a single agent administered every month or as continuous alternating inhaled therapy (CAIT), in which two (or more) antibiotics are alternated monthly. A double-blind trial indicated that three cycles of 28-day inhaled aztreonam (alternating with 28-day open-label TIS) are better tolerated and more efficacious in CF patients as compared with the intermittent use of TIS alone; however, the small sample size limits definitive conclusions [15]. Another study reported that the majority of patients with CF who switched from tobramycin to LIS had an improvement in FEV_1_ percentage predicted values [46].

All these studies emphasize that the use of continuous therapy with different combinations of inhaled antibiotics has grown over time. Therefore, it is not surprising that the prevalence of CF patients receiving more than one inhaled anti-*P. aeruginosa* antibiotic class increased from 2009 to 2012, as reported by the United States Cystic Fibrosis Foundation Patient Registry (CFFPR) [97,98]. Comparing these sequential treatments with inhaled aztreonam and tobramycin to tobramycin monotherapies, Estrella Rojo-Molinero and co-authors found that alternating antibiotics produced an increase in antibiofilm activity against the laboratory and clinical strains of *P. aeruginosa* [99]. Moreover, combinations of antibiotics against *P. aeruginosa* are commonly used to generate synergistic antibacterial activity and reduce drug resistance [100]. Finally, while CF patients may benefit from prolonged courses of inhaled antibiotics, there is currently little evidence to support the benefits of chronic maintenance therapy for bacteria other than *P. aeruginosa* [14].

## 6. Inhaled Antibiotic Therapy for Preventing Pulmonary Exacerbations

Pulmonary exacerbations are frequent clinical events in people with CF [30,101]. Although several criteria have been proposed for the definition of pulmonary exacerbations, there is still no consensus on the definition of a pulmonary exacerbation. Clinical manifestations of acute exacerbations are different depending on the age of the CF patient and disease stage, and the diagnosis of respiratory exacerbations remains a challenge, especially in young children [102]. Inhaled antibiotics play a crucial role in preventing acute exacerbations. Large numbers of CF patients receiving tobramycin and aztreonam by means of continuous alternating therapy continue to experience the exacerbations associated with lung function decline, highlighting the need for developing new and more effective therapies based on inhaled antibiotics [98]. Associating inhaled antibiotics with standard intravenous therapy has also been studied in patients with CF (6–21 years) who were hospitalized due to pulmonary exacerbations [103]. This study did not support the use of inhaled and parenteral antimicrobial therapies for the treatment of respiratory exacerbations. More recently, a single-center, randomized, open-label, cross-over pilot study regarding *P. aeruginosa* infected CF adult patients (*n* = 16) with acute pulmonary exacerbations, showed that the combination of AZLI and intravenous colistimethate resulted in greater improvements in lung function and quality of life, as compared with the use of intravenous antibiotics alone [104]. These data underscore the need for additional trials regarding the use of inhaled antibiotics for pulmonary exacerbations [104].

**Figure 2:**
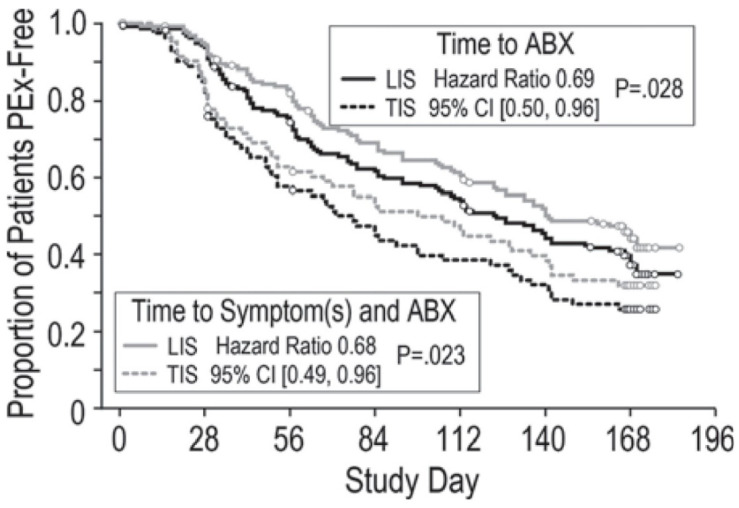
Difference in time to PEx as first antipseudomonal antibiotic (ABX) treatment (black lines) and ABX treatment in the presence of symptoms (gray lines) from Fischer R. et al., *Pediatr. Pulmonol.* **2016**, *Suppl 45*, 359, doi:10.1002/ppul.23573.

The administration of inhaled antibiotics two or three times daily (depending on the drug) has been reported to be the best therapeutic strategy for preventing acute exacerbations [30]. Levofloxacin is the most recently approved inhaled antibiotic for CF patients and appears to be a promising effective alternative nebulized antibiotic for preventing pulmonary exacerbations. In fact, encouraging results have been obtained regarding its capability to prevent the frequency of acute exacerbations [64]. Specifically, it has been demonstrated that CF adults receiving LIS were at a lower risk of pulmonary exacerbations, as shown by antipseudomonal antibiotic (ABX) treatment (hazard ratio 0.69, (0.50, 0.96) *p* = 0.028) and ABX treatment in the presence of symptoms (0.68 (0.49, 0.96) *p* = 0.023) as compared to those treated with TIS (Figure 2). They also showed greater FEV_1_ change in percentage predicted values from baseline at the end of each 28-day treatment. 

These findings suggest that inhaled antibiotics, such as LIS administered under new treatment schedules (e.g., CAIT), could play a key role in preventing exacerbations and, with the introduction of this novel therapeutic strategy, the epidemiology and manifestation of respiratory exacerbations could be very different. However, few data are available regarding the effects of alternative therapeutic regimens by means of combining more than one inhaled antibiotic or using continuous rather than cycled inhaled antibiotics. Therefore, a better understanding of exacerbation prevention strategies is required in order to have long-term benefits for CF patients [105].

## 7. Airway Conditions and Clinical Efficacy of Inhaled Antibiotics

Adherence is considered an important determinant of the effectiveness of inhaled antibiotic treatment in CF patients [106]. Rates of adherence to inhaled antibiotics vary substantially between age groups and have been shown to be associated with the complexity of the treatment regimen, time and duration of nebulization, treatment burden and patient satisfaction [107].

In order to obtain the clinical efficacy of treatments, a key issue is to understand how the local environment changes the effect of antibiotic aerosols after their deposition in the respiratory tract of patients with CF. The mucus in CF patients has different viscoelastic properties, thickness, and composition as compared to the mucus in healthy donors. Biofilms, as well as airway mucus, are well known to act as a protective physical barrier to inhaled antibiotics [108]. Studies regarding azithromycin have shown that inhibition of biofilm formation seemed to have a positive effect on CF progression [109,110,111]. However, these barriers have scarcely been examined in the in vitro models used during the preclinical development stages of anti-microbial drug candidates. In this context, the alginate layer surrounding *P. aeruginosa* can absorb antibiotics, and polycationic antibiotics (tobramycin and colistin) may bind to secretory mucin by means of electrostatic interactions, with negatively charged mucin glycoproteins reducing the free drug levels [112,113,114]. Accordingly, Müller L et al. reported a decrease in tobramycin efficacy and diffusion when *P. aeruginosa* biofilms were exposed to mucus as compared to those grown in mucus-free environments, pointing out the crucial role played by mucus and biofilms in terms of the efficacy of antimicrobial compounds [115]. In contrast, the efficacy of colistin in the presence of mucus does not appear to be affected [115]. In addition, the effects of nebulized antibiotics on biofilms are different, and LIS exhibits stronger anti-biofilm activity as compared to aminoglycosides and aztreonam [116]. A recent study also demonstrated that the chemical modification of tobramycin by means of PEGylation had higher in vitro activity against *P. aeruginosa* biofilm formation as compared to tobramycin in a CF-like mucus barrier model [117]. However, few investigations have been conducted into how bacterial interactions may contribute to the inability of antimicrobial therapies to clear *P. aeruginosa* from the lungs of CF patients. In this complex scenario, a novel mechanism of antibiotic resistance to tobramycin, based on the polymicrobial interactions between *P. aeruginosa* and *S. aureus*, has recently been identified. An interaction between the *S. aureus* product secreted, staphylococcal protein A (SpA), and a *P. aeruginosa* exopolysaccharide (Psl) has been discovered; this leads to aggregation and increased tobramycin resistance in the *P. aeruginosa* isolates obtained from CF children [118].

Moreover, observations from studies during the past decade have changed the conventional view of microbial community composition in CF, which has traditionally focused on a limited number of certain opportunistic bacteria [119]. It has now been determined that a complex mixed community of microorganisms colonizes the lower airways, referred to as the CF lung microbiome. More importantly, the changes in the structure and activity of the microbiome influence patient clinical conditions and disease outcome [120]. The administration of systemic antibiotics has been demonstrated to elicit significant changes in the microbial composition [120,121,122]. Community diversity and richness of the different anaerobic species decrease under antibiotic exposure [123]. Accordingly, recent studies have suggested that inhaled antibiotics may cause a marked change in the lower airway microbiota composition in CF patients, and that these bacterial communities may influence treatment response [124]. Interestingly, Heirali et al. reported no major changes in the microbiome during a single 28-day on/off inhaled-aztreonam regimen in adults with chronic *P. aeruginosa* infection [125]. This study also showed that non-responder patients had increased levels of *Fusobacterium* before and after the initiation of the inhaled AZLI treatment. Moreover, Heirali et al. found that two operational taxonomic units (OTUs) assigned to *Prevotella* were more elevated in non-responders in the pre-sample assessment, while, when they examined the post-samples, they observed that responders had an increase in *Prevotella* richness. More recently, the same authors have reported that the microbiome in CF adults exhibited resilience to inhaled aztreonam perturbations, and that non-responders had a higher level of aztreonam-resistant bacteria [126].

Although microbiota analysis is currently not recommended in routine microbial surveillance [44], these findings have suggested that a better understanding of the role played by the CF lung microbiome on inhaled antibiotic efficacy may contribute to identifying novel therapeutic and diagnostic approaches to better tackle chronic, pulmonary infections in this patient population. Moreover, an improved understanding of the complex pulmonary environment could contribute to defining optimal treatment regimens target CF pathogens without any effect on others [98].

## 8. Conclusions

Even in the era of disease-modifying agents by means of modulators of the CF transmembrane regulator, the development of novel nebulized antibiotic therapies is likely to remain important, especially for CF patients with advanced lung disease. In fact, one of the under-achieved goals of treatment in CF is the prevention of infections and progressive lung damage. Inhaled antibiotic therapy has the capacity to achieve higher concentrations in the sputum for antibacterial efficacy, while mitigating the toxicity associated with systemic treatment. Additionally, antibiotic therapy has also shown encouraging results for the treatment of infections in people with CF. Unfortunately, there are a limited number of inhaled antibiotics available on the market. Colistin, tobramycin, aztreonam lysine and, more recently, levofloxacin have been approved in different countries for CF patients. Adult patients with CF, receiving levofloxacin, were at lower risk of pulmonary exacerbations. In particular, the alternate continuous use (on/on) of inhaled antibiotics has become increasingly popular—especially among patients infected with *P. aeruginosa* with impairment of lung function. In contrast, the management of infections caused by all the classic pathogens other than *P. aeruginosa* has not been well supported by the results of clinical trials. In addition, despite considerable progress in understanding the effects of pulmonary exacerbation on outcomes in CF, there are multiple important knowledge gaps as regards the management of pulmonary exacerbations, and evidence from more interventional studies is needed to guide practice decisions.

Intensive treatments are highly recommended; however, measures to prevent new pulmonary exacerbations are even more relevant. Thus, the use of inhaled antibiotics, including the new antibiotic levofloxacin, administered in the early stage and under a CAIT regimen, could play a critical role in preventing exacerbations in CF patients. However, these preliminary observations need significant clinical validation before being considered viable therapeutic options for pulmonary exacerbations. Moreover, additional studies regarding the role played by the mucus environment on the inhaled antibiotic susceptibility of *P. aeruginosa* biofilms and on the characterization of the respiratory microbiome are needed to optimize the therapeutic management of CF infections with nebulized antibiotics.

## Figures and Tables

**Table 1:** Aerosolized antibiotics for the treatment of *P. aeruginosa* infection in CF patients.

Antibiotics	Type of Antibiotic	Mechanism of Action	Formulations	Trade Name	Nebulization Time	Dosage	Frequency
Tobramycin	Aminoglycosides	Inhibition of protein synthesis	Solution for nebulization	Tobramycin	15 min	300 mg/5 mL	Twice daily
				Tobi	15 min	300 mg/5 mL	Twice daily
				Bramitob	15 min	300 mg/4 mL	Twice daily
				Vantobra	4 min	170 mg/1.7 mL	Twice daily
Aztreonam lysine	Monobactams	Inhibition of bacterial cell wall synthesis	Solution for nebulization	Cayston	2–3 min	75 mg/1 mL	Three times daily
Levofloxacin	Fluoroquinolones	DNA gyrase and topoisomerase IV	Solution for nebulization	Quinsair	5 min	240 mg/3 mL	Twice daily
Colistimethate sodium *	Polymyxins	Disruption of bacterial cell membrane	Solution for nebulization	Promixin	3 min	80 mg/3 mL	Twice/Three times daily
				Colfinair	3–4 min	80 mg/3 mL	Twice/Three times daily

* Other colistin-based medical products may be used in other countries.
